# Dietary Vitamin A Impacts Refractory Telogen

**DOI:** 10.3389/fcell.2021.571474

**Published:** 2021-02-05

**Authors:** Liye Suo, Christine VanBuren, Eylul Damla Hovland, Natalia Y. Kedishvili, John P. Sundberg, Helen B. Everts

**Affiliations:** ^1^Department of Human Nutrition, The Ohio State University, Columbus, OH, United States; ^2^Department of Nutrition and Food Sciences, Texas Woman’s University, Denton, TX, United States; ^3^Department of Biochemistry and Molecular Genetics, Schools of Medicine and Dentistry, University of Alabama at Birmingham, Birmingham, AL, United States; ^4^The Jackson Laboratory, Bar Harbor, ME, United States

**Keywords:** vitamin A, retinoid metabolism, hair cycle, stem cells, telogen

## Abstract

Hair follicles cycle through periods of growth (anagen), regression (catagen), rest (telogen), and release (exogen). Telogen is further divided into refractory and competent telogen based on expression of bone morphogenetic protein 4 (BMP4) and wingless-related MMTV integration site 7A (WNT7A). During refractory telogen hair follicle stem cells (HFSC) are inhibited. Retinoic acid synthesis proteins localized to the hair follicle and this localization pattern changed throughout the hair cycle. In addition, excess retinyl esters arrested hair follicles in telogen. The purpose of this study was to further define these hair cycle changes. BMP4 and WNT7A expression was also used to distinguish refractory from competent telogen in C57BL/6J mice fed different levels of retinyl esters from two previous studies. These two studies produced opposite results; and differed in the amount of retinyl esters the dams consumed and the age of the mice when the different diet began. There were a greater percentage of hair follicles in refractory telogen both when mice were bred on an unpurified diet containing copious levels of retinyl esters (study 1) and consumed excess levels of retinyl esters starting at 12 weeks of age, as well as when mice were bred on a purified diet containing adequate levels of retinyl esters (study 2) and remained on this diet at 6 weeks of age. WNT7A expression was consistent with these results. Next, the localization of vitamin A metabolism proteins in the two stages of telogen was examined. Keratin 6 (KRT6) and cellular retinoic acid binding protein 2 (CRABP2) localized almost exclusively to refractory telogen hair follicles in study 1. However, KRT6 and CRABP2 localized to both competent and refractory telogen hair follicles in mice fed adequate and high levels of retinyl esters in study 2. In mice bred and fed an unpurified diet retinol dehydrogenase SDR16C5, retinal dehydrogenase 2 (ALDH1A2), and cytochrome p450 26B1 (CYP26B1), enzymes and proteins involved in RA metabolism, localized to BMP4 positive refractory telogen hair follicles. This suggests that vitamin A may contribute to the inhibition of HFSC during refractory telogen in a dose dependent manner.

## Introduction

The hair follicle has a self-cycling ability. It goes through a regeneration phase (anagen), a degenerative phase (catagen) involving the apoptosis and loss of the lower two-thirds of the hair follicle, a resting phase (telogen), and a release phase (exogen) (Chase et al., 1951; Milner et al., 2002). The transition from telogen to anagen requires the activation and proliferation of stem cells of the hair follicle bulge (HFSC) (Cotsarelis et al., 1990). Telogen hair follicles can be divided into two stages, refractory and competent telogen, based on the expression of bone morphogenetic protein 2 and 4 (BMP2 and BMP4) (Plikus et al., 2008). During refractory telogen BMP2, BMP4, BMP6, and fibroblast growth factor 18 (FGF18) keep HFSC in the quiescent state (Chen and Chuong, 2012; Hsu and Fuchs, 2012). During competent telogen these inhibitory factors are themselves inhibited. Transforming growth factor beta (TGFB) and the BMP inhibitor Noggin inhibit BMP signaling to trigger anagen initiation (Botchkarev et al., 1999, 2001; Oshimori and Fuchs, 2012). WNT7A and WNT7B are also directly inhibited by BMP signaling and relief of this inhibition is important for the initiation of the new hair cycle (Kandyba et al., 2013; Kandyba and Kobielak, 2014). According to this theory, anagen initiation can only occur if the hair follicle is in competent telogen. BMP6 and FGF18 are secreted from inner bulge cells, which are marked by keratin 6 (KRT6) (Hsu et al., 2011). This KRT6 layer has also been called suprabasal bulge cells (Wang et al., 2016), companion layer of the club hair, and trichilemmal keratin (Higgins et al., 2009). Hsu et al. (2011) showed that these cells are derived from the outer root sheath (ORS) and contain many stem cell regulators. Thus, they are neither companion layer nor simply differentiated ORS. The term inner bulge cells will be used throughout to define this layer. These inner bulge KRT6 positive cells also anchor the hair follicle to the club hair and are involved in shedding hair during late exogen (Higgins et al., 2009, 2011).

Retinoids is a general term for both synthetic and natural forms of vitamin A. The dietary form of vitamin A is retinyl esters. Retinyl esters are important for healthy hair (Everts, 2012); since both too much or too little retinyl esters leads to alopecia (hair loss) in humans and other mammals (Wolbach and Howe, 1925; Anzano et al., 1979; Berth-Jones and Hutchinson, 1995; Bleasel et al., 1999; Shih et al., 2009; Baldwin et al., 2012; Ocon et al., 2012; Duncan et al., 2013; Everts et al., 2013; Suo et al., 2015). Synthetic retinoids, such as acitretin and etretinate, cause alopecia at doses between 25 and 75 mg/d (Gupta et al., 1989; Murray et al., 1991; Katz et al., 1999). This retinoid-associated alopecia is dose-dependent and hair loss is reversible after significant reduction of retinoid dose or cessation of the systemic therapy (Gupta et al., 1989; Murray et al., 1991; Berth-Jones and Hutchinson, 1995; Katz et al., 1999). Retinoid-induced alopecia results in an arrest of hair follicles in telogen combined with an anchorage defect in the follicle during telogen (Berth-Jones and Hutchinson, 1995). Retinoid-induced alopecia also reduced anagen in cultured human hair follicles by inducing TGFB2 and triggering catagen (Foitzik et al., 2005). The mechanisms by which vitamin A regulates HFSC during the transition from telogen to anagen are still unknown.

The active form of vitamin A is retinoic acid (RA). RA is synthesized at or near the site of action (Duester, 2013; Obrochta et al., 2014), by a few key enzymes (Napoli, 2012). In the cell, retinol is either esterified to retinyl ester for storage by the enzymes lecithin:retinol acyltransferase and diglyceride acyltransferase 1 (DGAT1) (MacDonald and Ong, 1988; Orland et al., 2005); or retinol is reversibly oxidized to retinal by retinol dehydrogenases (Rexer and Ong, 2002; Napoli, 2012; Kedishvili, 2013). Five retinol dehydrogenases localize to the skin including: dehydrogenase reductase SDR family member 9 (DHRS9) (Rexer and Ong, 2002; Markova et al., 2003; Nadauld et al., 2005; Everts et al., 2007), retinol dehydrogenase 1/16 (RDH1/16) (Jurukovski et al., 1999; Karlsson et al., 2003), retinol dehydrogenases short-chain dehydrogenase/reductase family 16C members 5 and 6 (SDR16C5 and SDR16C6) (Belyaeva et al., 2012; Adams et al., 2017), and retinol dehydrogenase 10 (RDH10) (Wu et al., 2002; Lee et al., 2011). Retinal is further irreversibly oxidized to RA by retinal dehydrogenases 1–3 (ALDH1A1–3) (Napoli, 1999). RA binds cellular retinoic acid binding proteins (CRABPs) that transport RA throughout the cell (Ong, 1987). CRABP2-bound RA is transported into the nucleus and delivered to retinoic acid receptors (RARA, RARB, and RARG) (Dong et al., 1999; Sessler and Noy, 2005). RA and its receptors activate the transcription of over 500 genes involved in proliferation and differentiation (Giguere et al., 1987; Balmer and Blomhoff, 2002). Both CRABP1 and CRABP2 also transport RA into the endoplasmic reticulum to cytochrome P450 family members (CYP26A1, CYP26B1, and CYP26C1) that degrade RA and protect cells from vitamin A toxicity (Boylan and Gudas, 1992; Chen et al., 2003; Isoherranen and Zhong, 2019).

In a previous study in C57BL/6J (B6) mice, retinyl esters dose dependently altered the hair cycle (Everts et al., 2013). Feeding B6 mice an unpurified diet containing up to 28 IU retinyl esters/g diet during breeding and then excess levels of retinyl esters (56 IU/g diet) at 12 weeks of age resulted in more hair follicles in telogen (Study 1). However, when B6 mice were fed adequate retinyl esters (4 IU/g diet) during breeding and then excess levels of retinyl esters at 6 weeks of age there was no difference in the hair cycle (Study 2). Excess RA in *Dgat1*^*tm1Far*^ null mice also altered the hair cycle by both lengthening the first anagen and shortening the second telogen (Shih et al., 2009). These effects were partially reversed when the mice were fed a retinyl ester deficient diet. Excess RA in *Cyp26b1*^*tm1Hh*^ null mice blocked embryonic hair development; but the hair cycle could not be examined due to perinatal lethality (Okano et al., 2012). Conditional deletion of *Cyp26b1* in the dermal papilla and dermis (*En1Cre;Cyp26b1^*tm1Hh*^*) reduced zig-zag hairs, but did not block hair development. Recently, reduced retinal synthesis in *Del(4Sdr16c5-Sdr16c6)1Nyk* double null mice altered the hair cycle (Wu et al., 2019). These mice entered anagen at a younger age than controls; however, a complete analysis of the hair cycle was not performed. Initial localization studies done before the discovery of two telogen stages observed ALDH1A2 and CRABP2 localized to some, but not all telogen hair follicles (Everts et al., 2007). In contrast, DHRS9 did not localize to the hair follicle until anagen. This study was done to better define the effects of retinyl ester on the hair cycle.

## Materials and Methods

### Animals

#### Mice

The Jackson Laboratory (Bar Harbor, ME, United States) Institutional Animal Care and Use Committee approved all procedures. C57BL/6J (Stock number 664; hereafter referred to as B6) mice (The Jackson Laboratory, Bar Harbor, ME, United States) were used.

#### Hair Cycle Localization Study

B6 Dams and female pups were fed the NIH 31 diet (LabDiet 5K52, Purina Mills, St. Louis, MO, United States) throughout this study. Only females were used because they fight less, which reduces wounds. Wounds by themselves will initiate anagen, which would interfere with this study (Sundberg et al., 2018). Mice were sacrificed and skin collected at 70, 77, 80, 83, 84, 85, 86, 88, 90, 91, 93, 97, and 100 days of age to obtain hair follicles in all stages of the hair cycle without manipulation. Skin from one to three regions of the back (interscapular, thoracolumbar, and lumbar regions) from one to five mice were collected at each time point, Skin was fixed overnight in Fekete’s solution (61% ethanol, 3.2% formaldehyde, and 0.75 N acetic acid), and processed routinely for histology and immunohistochemistry (Everts et al., 2007). Approximately 60 hair follicles were analyzed per section of skin collected. The guide suggested by Muller-Rover et al. (2001) was used to stage hair follicles based on histology (Muller-Rover et al., 2001).

#### Study 1

**FIGURE 1:**
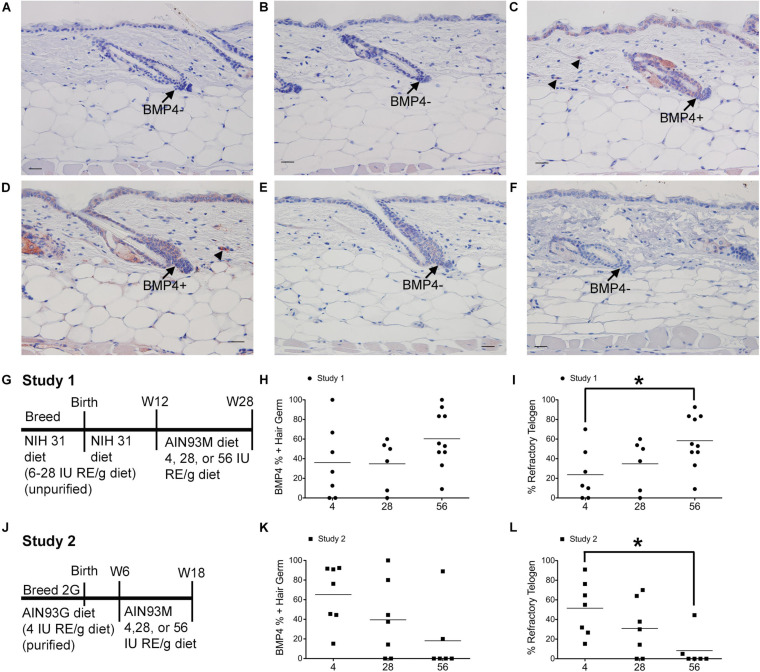
Dietary vitamin A impacts the percent of BMP4 positive refractory telogen hair follicles. C57BL/6J wild type Dams and female pups were fed either the NIH 31 diet containing 6–28 IU retinyl esters (RE)/g diet **(A–C,G–I)** or the AIN93G diet containing 4IU retinyl acetate/g diet **(D–F,J–L)** for two generations (2G). At either 12 **(A–C,G–I)** or 6 **(D–F,J–L)** weeks (W) of age these mice were switched to the AIN93M diet containing 4 (**A,D**, *n* = 7–8), 28 (**B,E**, *n* = 9–10), or 56 (**C,F**, *n* = 6–10) IU retinyl acetate/g diet for either 16 **(A–C,G–I)** or 12 **(D–F,J–L)** weeks. **(G,J)** show the feeding protocol for the two studies. Immunohistochemistry (IHC) was performed with antibodies against BMP4 (Abcam) in all skin containing telogen hair follicles. Telogen hair germs were counted as either positive or negative for BMP4. An average of 37 telogen hair germs per mouse were scored (3–70). The fraction of BMP4 positive telogen hair germs was calculated by dividing the number of BMP4 positive telogen hair germs by the total number of telogen hair germs. This fraction was then multiplied by either 100 to get the percent of positive hair germs **(H,K)** or the percentage of telogen follicles calculated in the original diet studies (Everts et al., 2013) to determine the percentage of hair follicle in refractory telogen **(I,L)**. A 2 × 3 ANOVA revealed an interaction between study and diet. *p < 0.05, by a one-way ANOVA performed on the interaction variable followed by Tukey *post-hoc* test. Bar = 25.2 μm. Arrow, hair germ; arrowhead, dermal cell.

B6 Dams and female pups were fed the NIH 31 diet (LabDiet 5K52, Purina Mills, St Louis, MO) until 12 weeks of age, and then switched to an American Institute of Nutrition (AIN)93 maintenance diet containing 4 (*n* = 8), 28 (*n* = 9), or 56 (*n* = 10) IU retinyl acetate/g diet (Research Diets, Inc., New Brunswick, NJ, United States) for 16 weeks (Figure 1G) (Everts et al., 2013). All mice with telogen hair follicles were analyzed. Only females were used to avoid wounds (Sundberg et al., 2018). In addition, female mice are more prone to central centrifugal cicatricial alopecia (CCCA), which was the focus of the initial diet study (Sundberg et al., 1994a). The first study was done to test the hypothesis that high retinyl esters worsens CCCA in B6 mice, since we saw an increased expression of RA synthesis proteins in both patients with CCCA and the B6 mouse model. The doses were chosen based on recommendations and measuring retinyl ester levels in the diet fed mice at The Jackson Laboratory production facility. The AIN and National Research Council recommend 4 IU/g diet; and 28 IU/g diet was the highest level measured in the standard diets at The Jackson Laboratory. We chose 56 as two times the highest level. However, this is not what we saw. None of the mice fed the highest level of retinyl esters had hair loss and they all had the majority of their hair follicles in telogen. There was an increased severity of disease in mice fed the 28 versus 4 IU diet. There was no specific reason for starting study one when the mice were 12 weeks old. These results lead us to perform a second study to reduce the level of retinyl esters and see how that impacted CCCA in the B6 mice. Breeding mice for three generations on the AIN93 growth diet reduced RA in several tissues (Obrochta et al., 2014) and reduced skin RA levels in *Dgat1*^*tm1Far*^ null mice (Shih et al., 2009).

#### Study 2

B6 Dams and their first and second-generation female pups were fed an AIN93 growth diet containing 4 IU retinyl acetate/g diet (Research Die, Inc., New Brunswick, NJ, United States). Second generation female pups were used in this study (Figure 1J). Six-week-old B6 mice were fed an AIN93 Maintenance diet containing 4 (*n* = 7), 28 (*n* = 10), or 56 (*n* = 6) IU retinyl acetate/g diet (Research Diets, Inc., New Brunswick, NJ, United States) for 12 weeks (Everts et al., 2013). At 6 weeks of age B6 mice are adults and have hair follicles in telogen. All mice with telogen hair follicles were analyzed.

### Immunohistochemistry (IHC)

**FIGURE 3:**
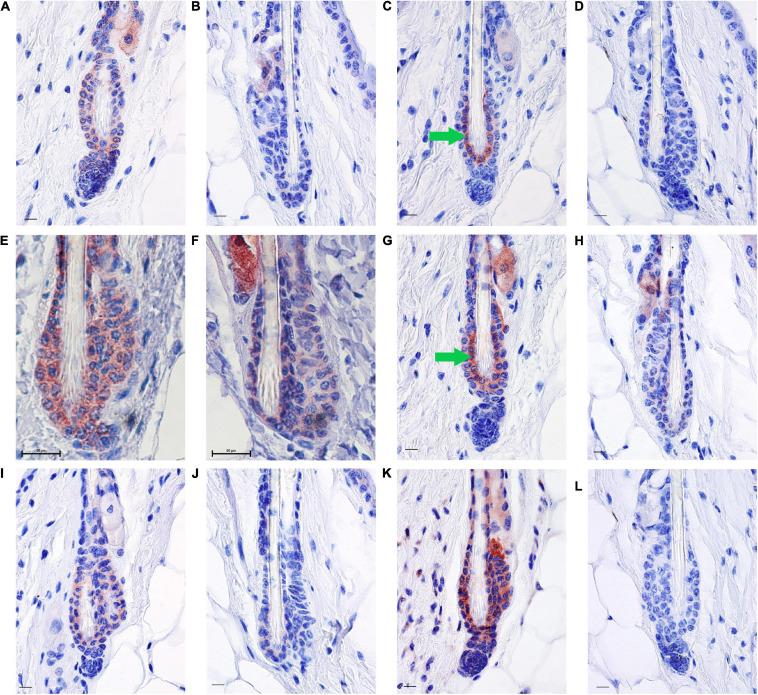
RA synthesis and degradation enzymes localize to BMP4 positive refractory telogen hair follicles. Skin from female C57BL/6J wild type mice fed an unpurified diet was collected at 70–101 days of age (*n* = 1–5 mice/time point). One to three regions of skin were collected per mouse. Immunohistochemistry (IHC) was performed with antibodies against BMP4 **(A,B)**, KRT6 **(C,D)**, SDR16C5 **(E,F)**, CRABP2 **(G,H)**, ALDH1A2 **(I,J)**, and CYP26B1 **(K,L)** using a red chromagen. Hair follicles that match the BMP4+ hair germ are on the left of each pair **(A,C,E,G,I,K)**, while hair follicles that match the BMP4– hair germ are on the right of each pair **(B,D,F,H,J,L)**. Details of these match ups are in Supplementary Figures 3, 4. An average of 60 hair follicles were examined per region of skin. Bar = 50 mm in **(E,F)**, and Bar = 10.1 μm in all other images. Images were cropped around the lower hair follicle. Whole images were minimally altered in Photoshop Elements using the exposure and balance settings in the “Quick” menu. Unaltered images are in Supplementary Figure 1. Green arrow, inner bulge cells.

Immunohistochemistry was performed in serial sections as described previously (Everts et al., 2007). Primary antibodies to the following proteins were purchased: BMP4 (Abcam, Cambridge, MA, United States; Vector Labs, Burlingame, CA, United States), WNT7A (Abcam, Cambridge, MA, United States), CYP26B1 (ACRIS, Rockville, MD, United States), KRT6 (Covance/BioLegend, Dedham, MA, United States), and RDH10 (Proteintech, Rosemont, IL, United States). Antibodies against ALDH1A2 and CRABP2 were made in the Ong laboratory (Everts et al., 2007). The antibody against SDR16C5 was made in the Kedishvili laboratory (Wu et al., 2019). The Vector anti-BMP4 antibody was made in mice. All other primary antibodies were made in rabbits. Biotinylated anti-rabbit or anti-mouse antibodies (Jackson ImmunoResearch Laboratories, Inc., West Grove, PA, United States) was used second, followed by a horseradish peroxidase conjugated anti-biotin antibody (Bethyl Laboratories, Inc., Montgomery, TX, United States). The red 3-amino-9-ethylcarbazole plus enhancers (AEC+; Dako, Carpinteria, CA, United States) substrate chromogen was used followed by counterstaining with Gils Hematoxylin III (Poly Scientific, Bay Shore, NY, United States) and mounting in aquamount (Dako, Carpinteria, CA, United States). Images were captured on either an Olympus BX51 microscope with the DP71 camera (Olympus, Tokyo, Japan) or Nikon Eclipse i80 microscope with the DS-Ri2 camera (Nikon Instruments Inc., Melville, NY, United States). Images were sized, cropped, and composite figures were created in Adobe Photoshop CS3 or Elements (San Francisco, CA, United States). Most figures are using unaltered images. For Figure 3 the whole images were minimally altered in Photoshop Elements using the exposure and balance settings in the “Quick” menu. Unaltered images are in Supplementary Figure 3.

Bone morphogenetic protein 4 positive and negative telogen hair germs and dermal cells were counted from mice fed various levels of vitamin A in studies 1 and 2. The percent of hair follicles in refractory telogen was calculated in two steps. First, the number of BMP4 positive telogen hair germs was divided by the total number of telogen hair follicles to get the fraction of telogen hair germs in refractory telogen. Second, this fraction was multiplied by the percentage of telogen follicles calculated in the original diet studies (Everts et al., 2013) because skin that contained only anagen or catagen hair follicles were not analyzed for BMP4. This percent of hair follicles in refractory telogen was subtracted from the total percent of telogen follicles to get the percent of follicles in competent telogen. An average of 37 telogen hair follicles per mouse were scored for BMP4 (3–70). Materials are available upon request.

### Co-immunofluorescence (Co-IFA)

Co-immunofluorescence was performed using a two-antibody co-localization system in frozen dorsal skin sections as previously described (Duncan et al., 2013). Primary antibodies against the following proteins were used with the indicated concentrations: ALDH1A2 (Rabbit, produced in the Ong laboratory), CRABP2 (Rabbit, produced in the Ong laboratory), CYP26B1 (Rabbit, ACRIS, Chapel Hill, NC, United States), BMP4 (Mouse, Vector Lab, Burlingame, CA, United States), and CD34 (Rat, BD Biosciences Pharmingen, San Jose, CA, United States). Secondary antibodies (anti-rat, anti-rabbit, anti-mouse, Invitrogen, Carlsbad, CA, United States) conjugated with Alexa Fluor 488 or Alexa Fluor 594 were used. Nuclei were stained with DAPI (Invitrogen, Carlsbad, CA, United States) for 1 min and subsequently the sections were washed and cover slipped with Prolong Gold anti-fade reagent (Molecular Probes, Eugene, OR, United States). Pictures were taken at 20X and 60X magnification (Nikon ACT-1, Tokyo) and processed in Adobe Photoshop CS4 (San Francisco, CA, United States).

### Co-IHC

Immunohistochemistry was first performed with antibodies against KRT6 and CRABP2 using AEC+ (red) as described above with additional blocking steps using the streptavidin, biotin blocking kit (Vector Labs, Burlingame, CA, United States). IHC was then performed with the Vector mouse anti-BMP4 antibody using the Mouse on Mouse ImmPRESS Peroxidase kit with some modifications (Vector Labs, Burlingame, CA, United States). First, we doubled the concentration of blocking reagent and incubated the slides for 2 h. Second, we diluted the ImmPRESS reagent 1:1 in the provided 2.5% normal horse serum. Slides were then treated with DAB Eq (brown) for 10 min, counterstained and mounted as described above. The number of hair follicles with positive KRT6 or CRABP2 plus or minus dermal BMP4 was counted. The percent of positive KRT6 or CRABP2 hair follicles with dermal BMP4 was then calculated separately from the percent of KRT6 or CRABP2 hair follicles without dermal BMP4. An average of 26 hair follicles (range of 2–54) were scored per mouse. Four to seven mice per diet were analyzed. Images were taken with the Nikon Eclipse i80 microscope with the DS-Ri2 camera (Nikon Instruments Inc., Melville, NY, United States). Images were sized, cropped, composite figures were created, and whole images were adjusted for brightness, contrast, and color balance in Adobe Photoshop CS3. Unaltered images are in Supplementary Figure 6.

### Statistical Analysis

Equal variance was first confirmed using Levene’s test of equality of error variances using SPSS version 24 (IMB SPSS Statistics, Chicago, IL, United States). Comparison among experimental groups was analyzed by univariate analysis of variance using a 2 × 3 full factorial model followed by Tukey *post-hoc* test when significant treatments effects were seen. When there was a significant interaction between study and diet a one-way ANOVA was performed on the interaction variable followed by Tukey *post-hoc* test. When equal variances were not seen, each study was analyzed by Kruskal–Wallis followed by Mann–Whitney *U* test. *P* value < 0.05 is defined as statistical significance.

## Results

**FIGURE 2:**
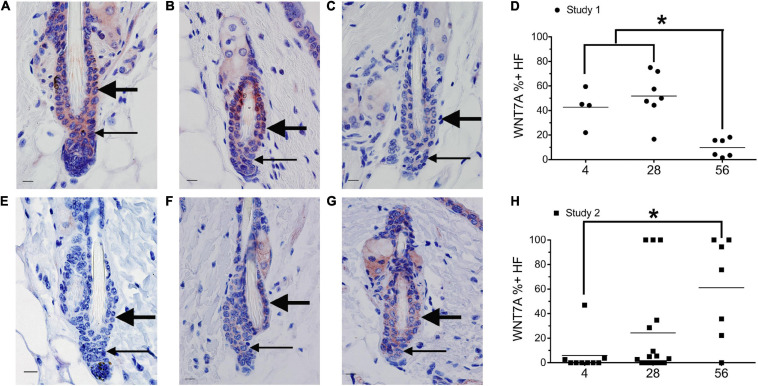
Dietary vitamin A regulates WNT7A. Mice from study 1 **(A–D)** or study 2 **(E–H)** were switched to the AIN93M diet containing 4 (**A,E**, *n* = 4–9), 28 (**B,F**, *n* = 7–16), or 56 (**C,G**, *n* = 6–7) IU retinyl acetate/g diet. Immunohistochemistry (IHC) was performed with antibodies against WNT7A in all skin containing telogen hair follicles. WNT7A positive and negative immunoreactivity in the club hair, bulge, and hair germ were counted together as hair follicles and the percent of positive hair follicles calculated **(D,H)**. An average of 29 (range of 1–72) telogen hair follicles per mouse were scored. *p < 0.05, by a one-way ANOVA followed by Tukey *post-hoc* test for study 1 and Kruskal–Wallis test followed by Mann–Whitney *U* test for study 2. Bar = 10.1 μm. Large arrow, club hair; small arrow, hair germ.

Mice from study 1 (Figure 1G) on the 56 IU diet had 98% of their hair follicles in telogen when analyzed histologically (Everts et al., 2013). To better define how dietary vitamin A altered the hair cycle, we first examined the expression of several regulators of the hair cycle. Using an antibody from Vector Labs we found that retinyl esters increased the number of dermal BMP4 positive cells in both studies (Supplementary Figure 1). Because we were not sure that the red immunoreactivity we were seeing in the dermis was really within the dermal cells, we repeated the study with a different antibody against BMP4. The Abcam antibody against BMP4 had immunoreactivity within the hair germ and epidermis in addition to the dermis, similar to BMP4-Lacz reporter mice (Figure 1; Plikus et al., 2008). Quantifying the percent of hair follicles with positive expression in the hair germ we saw a similar trend in study 1 as was seen with the other antibody (Figures 1A–C,H). However, in study 2 there was a trend for a drop in the percent of positive BMP4 hair germs as the amount of retinyl esters in the diet increased (Figures 1D–F,K). Using the BMP4 positive hair germ to indicate refractory telogen, we also calculated the percent of hair follicles in refractory telogen. BMP4 is high during refractory telogen and negative during competent telogen. Figure 1I shows that mice from study 1 fed the 56 IU diet had the majority of their hair follicles in refractory telogen. This was significantly greater than mice fed the 4 IU diet in study 1. Retinyl esters had no significant effect on the hair cycle in study 2 when analyzed histologically. Figure 1L shows that when these mice were fed the 56 IU diet significantly fewer hair follicles were in refractory telogen than mice fed 4 IU diet. WNT7A increases as hair follicles transition from competent telogen to anagen. Figure 2 shows that fewer WNT7A positive hair follicles were seen in both the 56 IU diet group of study 1 and the 4 IU diet group of study 2. This further supports the findings that these hair follicles are in refractory telogen.

**FIGURE 4:**
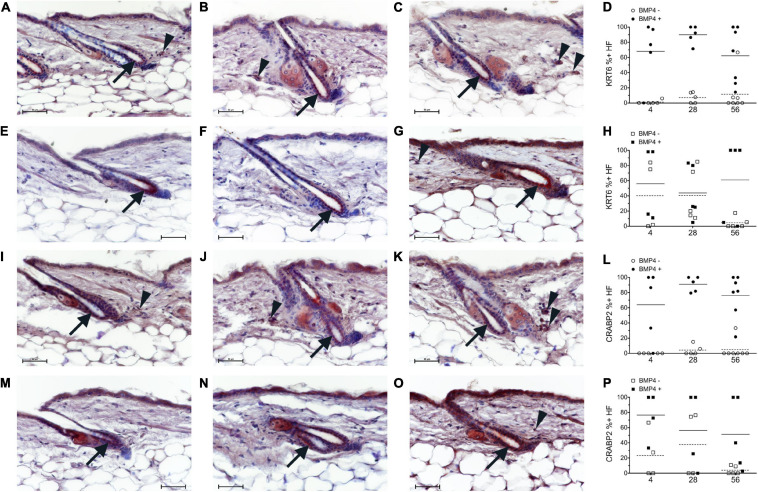
Breeding mice on AIN93G diet altered the localization pattern of KRT6 and CRABP2. Study 1 (**A–D,I–L**, circles) or study 2 (**E–H,M–P**, squares) mice were switched to the AIN93M diet containing 4 (**A,E,I,M** *n* = 4–5), 28 (**B,F,J,N**, *n* = 5), or 56 (**C,G,K,O**, *n* = 5–7) IU retinyl acetate/g diet. Co-Immunohistochemistry (IHC) was performed with antibodies against KRT6 and CRABP2 (red) with BMP4 (brown, Vector) in skin containing telogen hair follicles. Hair follicles with KRT6 and CRABP2 positive and negative localization in the inner bulge cells were counted separately for hair follicles with strong BMP4 positive cells in the dermis (Filled) and hair follicles without strong BMP4 positive cells in the dermis (open). The background brown in the arrector pili and dermal fat was ignored. The percent of positive KRT6 and CRABP2 hair follicles with and without BMP4 was calculated **(D,H,L,P)**. An average of 26 (range of 2–54) telogen hair follicles per mouse were scored. Whole images were minimally altered in Photoshop CS3. Unaltered images are in Supplementary Figure 6. Bar = 50 μm. Arrow, inner bulge cells; arrowhead, strong BMP4 positive dermal cells.

Next, we attempted to determine the localization pattern of vitamin A metabolism proteins during telogen using BMP4 to mark refractory telogen. Dorsal skin was collected from B6 mice fed an unpurified diet at 70–101 days of age and IHC performed in serial sections with antibodies against BMP4 (Abcam), KRT6, SDR16C5, RDH10, ALDH1A2, CRABP2, and CYP26B1. Using the Abcam antibody, all proteins localized to BMP4 positive telogen hair follicles (Figure 3 and Supplementary Figures 2, 3). SDR16C5 and ALDH1A2 were also seen in BMP4 negative telogen hair follicles, but at a lower level (Figure 3 and Supplementary Figures 2, 4). However, CRABP2 and CYP26B1 were barely detectable and KRT6 was negative in BMP4 negative follicles. CYP26B1 also localized to the dermis surrounding the BMP4 positive hair follicle, but not the BMP4 negative hair follicles. In addition, CRABP2 localized to the inner KRT6 positive bulge cells (Figures 3C,G, green arrows). RDH10 localized to all telogen hair follicles at similar levels (data not shown). This expression pattern contrasted the Co-IFA performed on frozen sections from a few mice from the retinyl ester diet studies using the Vector Labs antibody (Supplementary Figure 5). Thus, we ran Co-IHC with the Vector Labs BMP4 antibody, KRT6 and CRABP2 on samples from all of the diets in the retinyl ester feeding studies. We found that both KRT6 and CRABP2 localized to the inner bulge only when BMP4 was in the dermis in all but one mouse in study 1 (Figures 4A–D,I–L arrow and arrowhead and Supplementary Figure 6). However, when mice from study 2 were fed either the 4 or 28 IU diet KRT6 and CRABP2 localized to the inner bulge in both the presence and absence of dermal BMP4 (Figures 4E–H,M–P). All of the data are presented in the figures and Supplementary Data Sheet 1.

## Discussion

This study was done to better define the impact retinyl esters have on the hair cycle. In mice bred on an unpurified diet, excess (pharmacological) levels of retinyl esters lead to a greater percentage of hair follicles in refractory telogen. Regardless of retinyl ester level fed, KRT6 and CRABP2 localized to the inner bulge cells almost exclusively in refractory telogen in these mice. SDR16C5, ALDH1A2, and CYP26B1 also localized to the hair follicle at greater intensities during refractory telogen in mice bred and fed an unpurified diet. In contrast, when mice were bred on a purified diet containing recommended (physiological) levels of retinyl esters the percent of hair follicles in refractory telogen decreased as retinyl ester levels increased. In addition, KRT6 and CRABP2 localized to the inner bulge cells during both competent and refractory telogen in adult mice fed recommended (4IU) and high (28IU) levels of retinyl esters. These data suggest that vitamin A may regulate HFSC in a dose and time dependent manner.

Bone morphogenetic protein 2, BMP4, and BMP6 inhibit HFSCs during refractory telogen (Plikus et al., 2008; Hsu et al., 2011). BMP2 and 4 are secreted from adipocytes, while BMP6 localizes to KRT6 positive inner bulge cells (Hsu et al., 2011; Wang et al., 2016; Yi, 2017). KRT6 positive cells also express LIM homeobox protein 2 (LHX2), NFAT1C, and FGF18 during telogen that also inhibit HFSC to maintain telogen (Hsu et al., 2011; Kimura-Ueki et al., 2012; Yi, 2017). HFSC inhibition (quiescence) also requires forkhead box c1 (FOXC1) signaling during early anagen (Wang et al., 2016). FOXC1 directly increased NFATC1 and BMP6. RNA-seq in postnatal day 30 B6;SJL-Tg(*Krt1-15crePGR)22Cot/J/Foxc1^*tm1Blh*^* null mice identified increased *Aldh1a2* expression. Assay for transposase accessible chromatin then sequencing (ATAC-seq) analysis of the promoters of differentially expressed genes revealed a SMAD binding motif in the promoter of *Aldh1a2*, but not NFATC1 or FOXC1 binding sites. This suggests that BMP signaling may increase ALDH1A2 and RA synthesis during refractory telogen to contribute to HFSC quiescence.

Retinoic acid may also regulate HFSC quiescence. Excess RA in the *Cyp26b1*^*tm1Hh*^ null mice decreased *Lhx2* at embryonic day E16.5 and E18.5 along with altering several other HFSC regulatory genes and blocked hair follicle development (Okano et al., 2012). However, defective hair development was not seen in dermal papilla and dermal directed *En1^*tm2(cre)Wrst*^;Cyp26b1^*tm1Hh*^* null mice. The finding that CYP26B1 localized to the HFSC and inner bulge in addition to the dermis in this study suggests that reduced RA may be critical within the HFSC and/or inner bulge cells but not the dermal papilla. CRABP2 shuttles RA to both RARs and CYP26B1 (Dong et al., 1999; Nelson et al., 2016). This suggests that a low dose of RA may be necessary specifically in the inner bulge cells, while reduced RA is critical in the HFSC and dermis during refractory telogen. Further evidence for a role of RA in HFSC quiescence include: premature anagen entry with both reduced RA in *Del(4Sdr16c5-Sdr16c6)1Nyk* double null mice and excess RA in *Dgat1*^*tm1Far*^ null mice; and an increase of hair follicles in refractory telogen following excess retinyl esters in study 1, reduced retinyl esters in study 2, and pharmacological doses of synthetic retinoids in the clinic.

Retinoic acid synthesis proteins localizing to the KRT6 positive inner bulge cells during telogen is similar to what was seen in the inner cell layer and companion layer during anagen (Everts et al., 2007). ALDH1A3 localized to the KRT6 positive companion layer in the suprabulbar region, ALDH1A2 localized to the inner layer of the ORS in the isthmus and at a lower intensity in the companion layer in the suprabulbar region, and CRABP2 localized to the inner layer of the ORS in the infundibulum. The intensity of the immunoreactivity seems slightly higher in anagen than telogen, however, other methods besides IHC are needed to quantitate this small difference. The inner layer next to the anagen bulge expressed DHRS9, ALDH1A3, CRABP2, and RARA. This suggests that RA is important for these layers of the hair follicle throughout the hair cycle. Future studies are needed to determine the exact roles of RA in these cells; however, RA directly increases the expression of KRT6 (Navarro et al., 1995).

Keratin 6 positive inner bulge cells are also important for anchoring the club hair to the follicle, regulating the release of the club hair during exogen, and providing a reservoir of HFSC in the old hair follicle (Higgins et al., 2009, 2011; Wang et al., 2016). Microarray analysis revealed that *Cyp26b1* expression decreased in these inner bulge cells in late exogen verses early exogen (Higgins et al., 2011). A reduction of CYP26B1 would increase RA levels, suggesting that RA may also regulate hair shedding during exogen. High RA in *Dgat1*^*tm1Far*^ null mice lead to cyclical alopecia characterized by excess shedded hair during anagen, which is consistent with excess exogen or anagen effluvium (Shih et al., 2009). This alopecia was reduced when mice were fed a retinyl ester deficient diet. Thus, RA synthesis within the KRT6 positive inner bulge cells may impact exogen in addition to altering the telogen to anagen transition.

The cause of the elevated refractory telogen with low retinyl esters in study 2 is unclear. The two retinyl ester feeding studies had both different diets during breeding and different times when the diets where changed. This likely resulted in changes in retinyl esters at different stages of the hair cycle that were of different doses. Studies with both B6;SJL-Tg(*Krt1-15crePGR)22Cot/J, Foxc1^*tm1Blh*^* null mice and FGF18 injections revealed opposite effects based on the stage of the hair cycle the follicle was in at the time (Kimura-Ueki et al., 2012; Wang et al., 2016). The dissociation of KRT6 from BMP4 expression in study 2 suggests that the hair cycle stage may be important. Also note that most studies that examine HFSC regulation are done on mice fed an unpurified diet.

The dose of retinyl esters may also play a significant role in the U-shaped dose response curve seen in these studies. When low amounts of retinyl esters are consumed or not absorbed, vitamin A deficiency results in follicular hyperkeratosis and alopecia in both rats and humans (Wolbach and Howe, 1925; Anzano et al., 1979; Bleasel et al., 1999; Girard et al., 2006; Ocon et al., 2012). Excessive consumption of retinoids through retinyl ester supplementation or retinoid treatments also leads to alopecia (Gupta et al., 1989; Murray et al., 1991; Berth-Jones and Hutchinson, 1995; Katz et al., 1999; Shih et al., 2009). Vitamin A deficiency and pharmacological levels of RA also inhibit sebum production in the sebaceous gland (Zouboulis et al., 1993; Thiboutot et al., 2009; Barbieri et al., 2019). This is due to the hormesis effects of RA production (Napoli, 2020). As consumption of retinyl ester increases, the concentration of RA rises and provides increasing beneficial effects until the optimal concentration is reached. After this point, however, the benefits of retinyl ester supplementation decrease and then cease altogether. At high concentrations, RA is toxic and has deleterious effects on the organism. These data suggest that pharmacological doses of retinoids may arrest hair follicles in refractory telogen as a toxic effect of increased RA synthesis during this time. However, physiological doses of retinoids may play a greater role in anagen induction. Future studies are needed to tease out whether this is an effect of retinyl ester dose or timing of the retinyl ester.

The hair cycle impacts alopecia in different ways. In the context of central centrifugal cicatricial alopecia (CCCA, B6 dermatitis), arresting hair follicles in refractory telogen is a good thing. This is because CCCA in mice only occurs when hair follicles are in anagen (Sundberg et al., 2011). No mice fed the 56 IU diet in study 1 had alopecia in the previous study (Everts et al., 2013). Anagen hair follicles are also the target of immune attach in alopecia areata (AA) (Sundberg et al., 1994b). Thus, arresting hair follicles in refractory telogen can prevent some types of alopecia (CCCA, AA), while facilitating others such as telogen effluvium. Retinoids in different contexts and doses may impact the various forms of alopecia differently.

## Data Availability Statement

The original contributions presented in the study are included in the article/Supplementary Material, further inquiries can be directed to the corresponding author.

## Ethics Statement

The animal study was reviewed and approved by The Jackson Laboratory (Bar Harbor, ME, United States) Institutional Animal Care and Use Committee.

## Author Contributions

HE and JS conceptualized, acquired funding, provided resources, provided supervision, and reviewed and edited the manuscript. HE administered the project and curated the data. LS, CV, EH, and HE performed the analyses. LS wrote the original draft. CV wrote the first draft of the revision. NK provided resources. All authors contributed to the article and approved the submitted version.

## Conflict of Interest

The authors declare that the research was conducted in the absence of any commercial or financial relationships that could be construed as a potential conflict of interest.

## Abbreviations

Mouse genes/transcripts: first letter capitalized all subsequent letters lower case and all in italics
Proteins: all capital letters no italics
ANOVA: analysis of variance
BMP2, 4, 6: bone morphogenic protein 2, 4, 6
HFSC: hair follicle stem cell
SDR16C5, 6: retinol dehydrogenase short-chain dehydrogenase/reductase family 16C members 5 and 6
RDH10: retinol dehydrogenase 10
ALDH1A1, 2, 3: aldehyde dehydrogenase family member 1A1, 2, 3
CRABP2: cellular retinoic acid binding protein II
CYP26A1, B1: C1, cytochrome P450 family member 26A1, B1, C1
B6: C57BL/6J
FGF18: fibroblast growth factor 18
KRT6: keratin 6
ORS: outer root sheath
TGFB2: transforming growth factor beta 2
RA: retinoic acid
DGAT1: diglyceride acyltransferase 1
DHRS9: dehydrogenase/reductase SDR family 9
RDH1/16: retinol dehydrogenase 1/16
IU: international unit
NIH: national institutes of health
AIN: American Institute of Nutrition
IHC: immunohistochemistry
LHX2: LIM homeobox protein 2
NFAT1C: nuclear factor of activated T cells cytoplasmic calcineurin dependent 1
FOXC1: forkhead box c1
ATAC-seq: Assay for transposase accessible chromatin then sequencing
SMAD, RARA, RARB, RARG: retinoic acid receptors alpha, beta and gamma
CCCA: central centrifugal cicatricial alopecia
AA: alopecia areata.
