# Supplementary material for: Dietary Vitamin A Impacts Refractory Telogen
Source: Front Cell Dev Biol. 2021 Feb 5;9:571474. doi: 10.3389/fcell.2021.571474 (PMC7892905; doi:10.3389/fcell.2021.571474)
Supplement: Supplementary file 1 [file Data_Sheet_1.PDF]

Supplementary Material

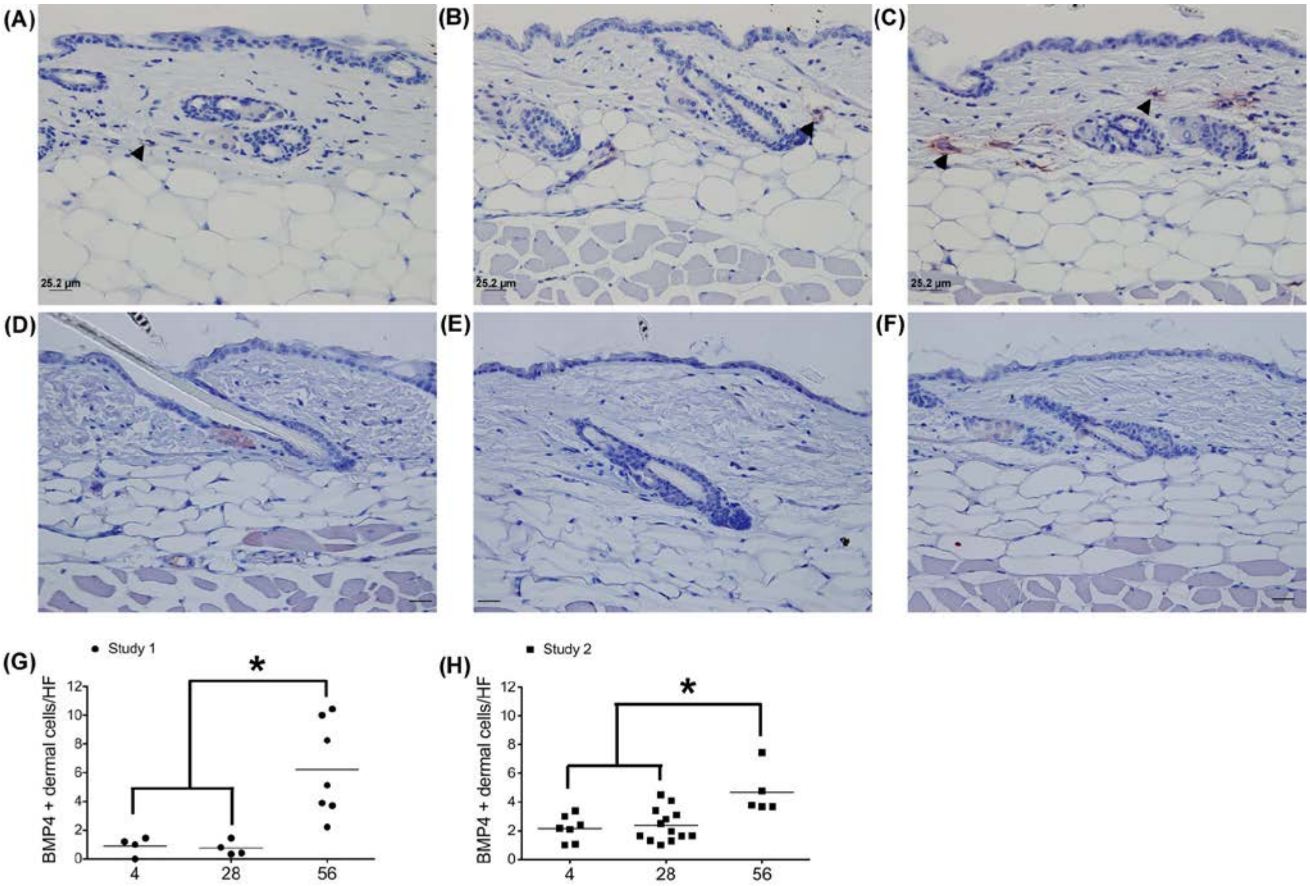

Supplemental Figure 1: Dietary vitamin A impacts dermal BMP4. Mice from study 1 (A-C, G) or study 2 (D-F, H) were switched to the AIN93M diet containing 4 (A, D, n=5-9), 28 (B, E, n=7-16), or 56 (C, F, n=6-7) IU retinyl acetate/g diet. Immunohistochemistry (IHC) was performed with antibodies against BMP4 (Vector) in all skin containing telogen hair follicles. BMP4 positive dermal cells around each hair follicle were counted and the number of BMP4 dermal cells per hair follicles calculated (G, H). An average of 28 (range of 2-78) telogen hair follicles per mouse were scored. * $p < 0.05$, by Kruskal-Wallis followed by Mann-Whitney U test for study 1 and ANOVA followed by Tukey *post-hoc* test for study 2. Bar = 25.2 um. Arrowheads = BMP4 positive dermal cells.

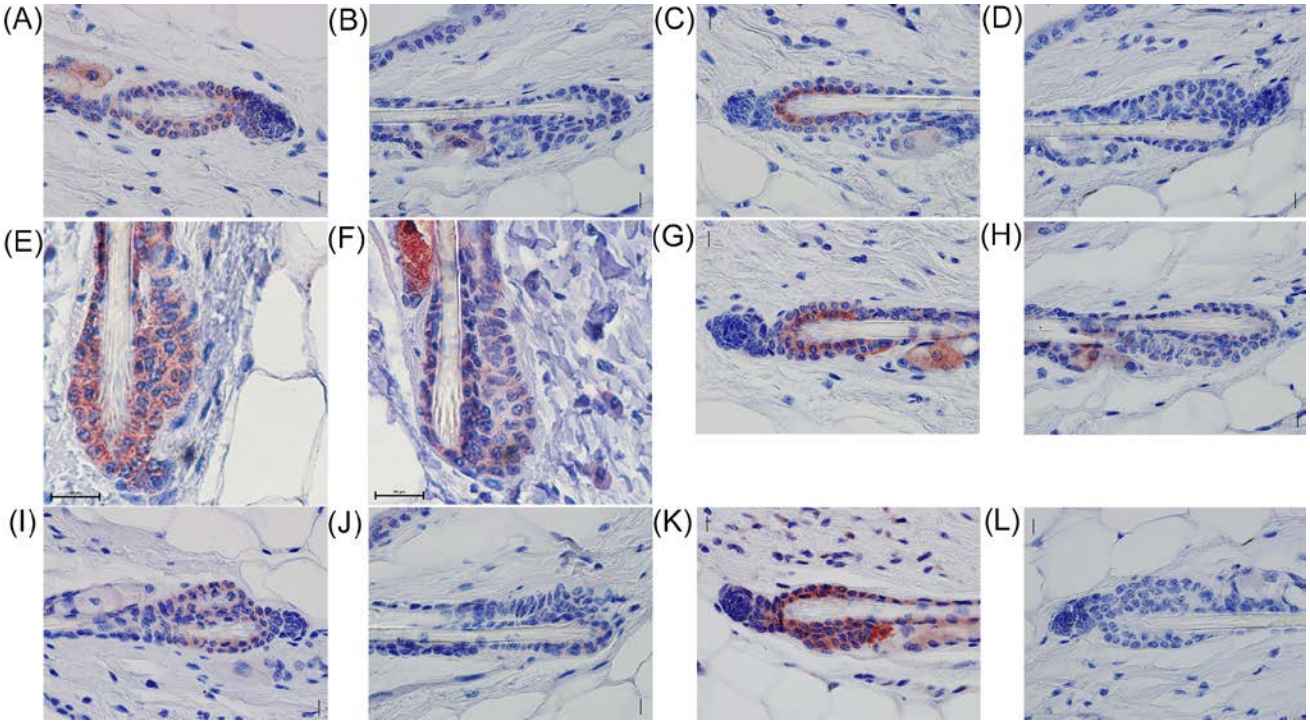

Supplemental Figure 2: RA synthesis and degradation enzymes localize to BMP4 positive refractory telogen hair follicles unaltered images. Skin from female C57BL/6J wild type mice was collected at 70-101 days of age (n=1-5 mice/time point). One to three regions of skin were collected per mouse. Immunohistochemistry (IHC) was performed with antibodies against BMP4 (A,B), KRT6 (C,D), SDR16C5 (E,F), CRABP2 (G,H), ALDH1A2 (I,J), and CYP26B1 (K,L) using a red chromagen. An average of 60 hair follicles were examined per region of skin. Bar = 50 mm in E and F, and Bar = 10.1 µm in all other images.

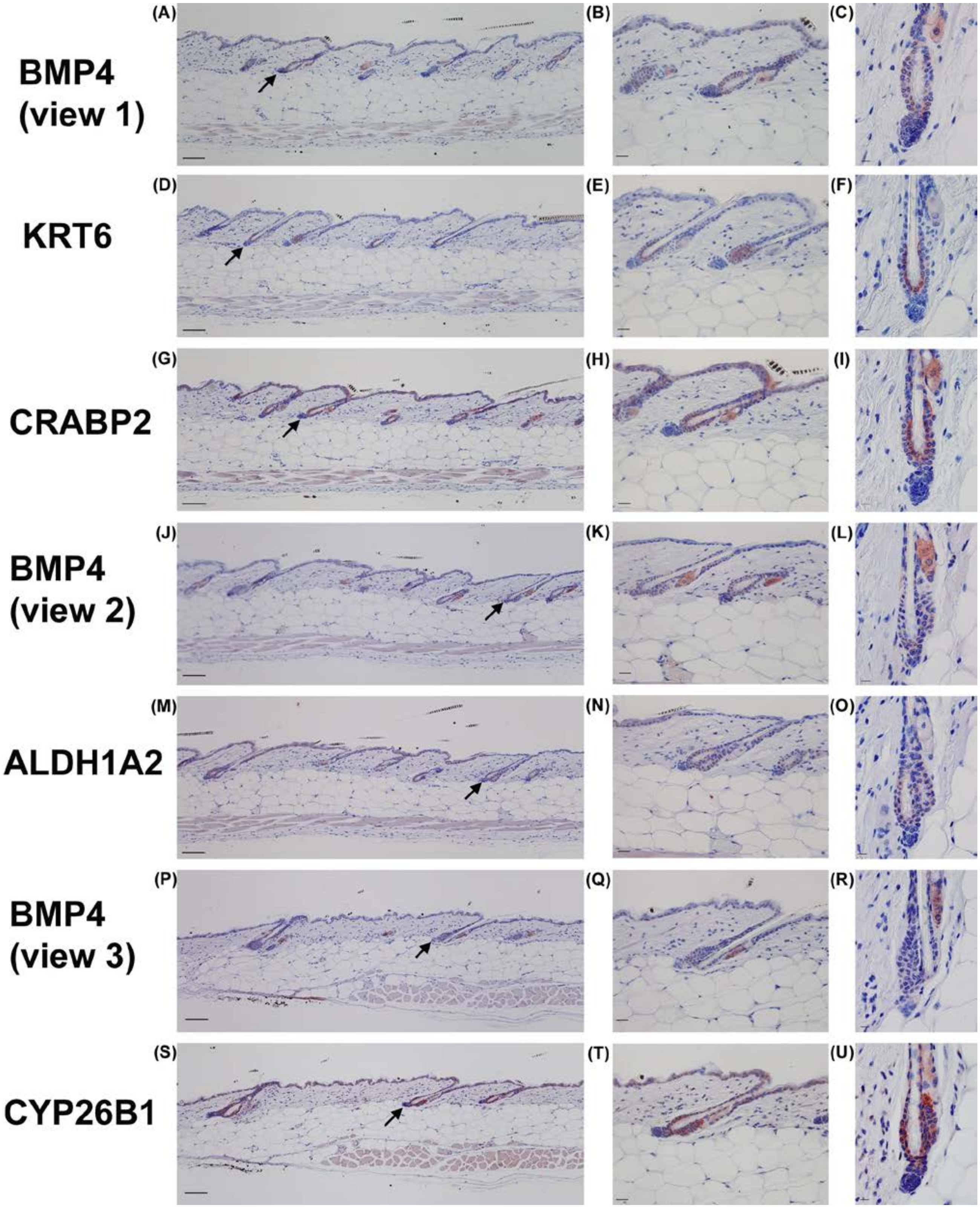

Supplemental Figure 3: Alignment of BMP4 positive hair follicles with vitamin A metabolism proteins. Skin from female C57BL/6J wild type mice was collected at 70-101 days of age (n=1-5 mice/time point). One to three regions of skin were collected per mouse. Immunohistochemistry (IHC) was performed on serial sections with antibodies against BMP4 (A-C, J-L, P-R), KRT6 (D-F),

CRABP2 (G-I), ALDH1A2 (M-O), and CYP26B1 (S-U) using a red chromagen. An average of 60 hair follicles were examined per region of skin. Bar = 100 um in A, D, G, J, M, P, and S, and Bar = 25.2 um in B, E, H, K, N, Q, and T, Bar = 10.1 µm in C, F, I, L, O, R, and U. Arrow points to the hair follicle used in the higher magnifications. The hair follicles with KRT6 and CRABP2 match the hair follicle with BMP4 view 1. The hair follicle with ALDH1A2 matches the hair follicle with BMP4 view 2. The hair follicle with CYP26B1 matches the hair follicle with BMP view 3.

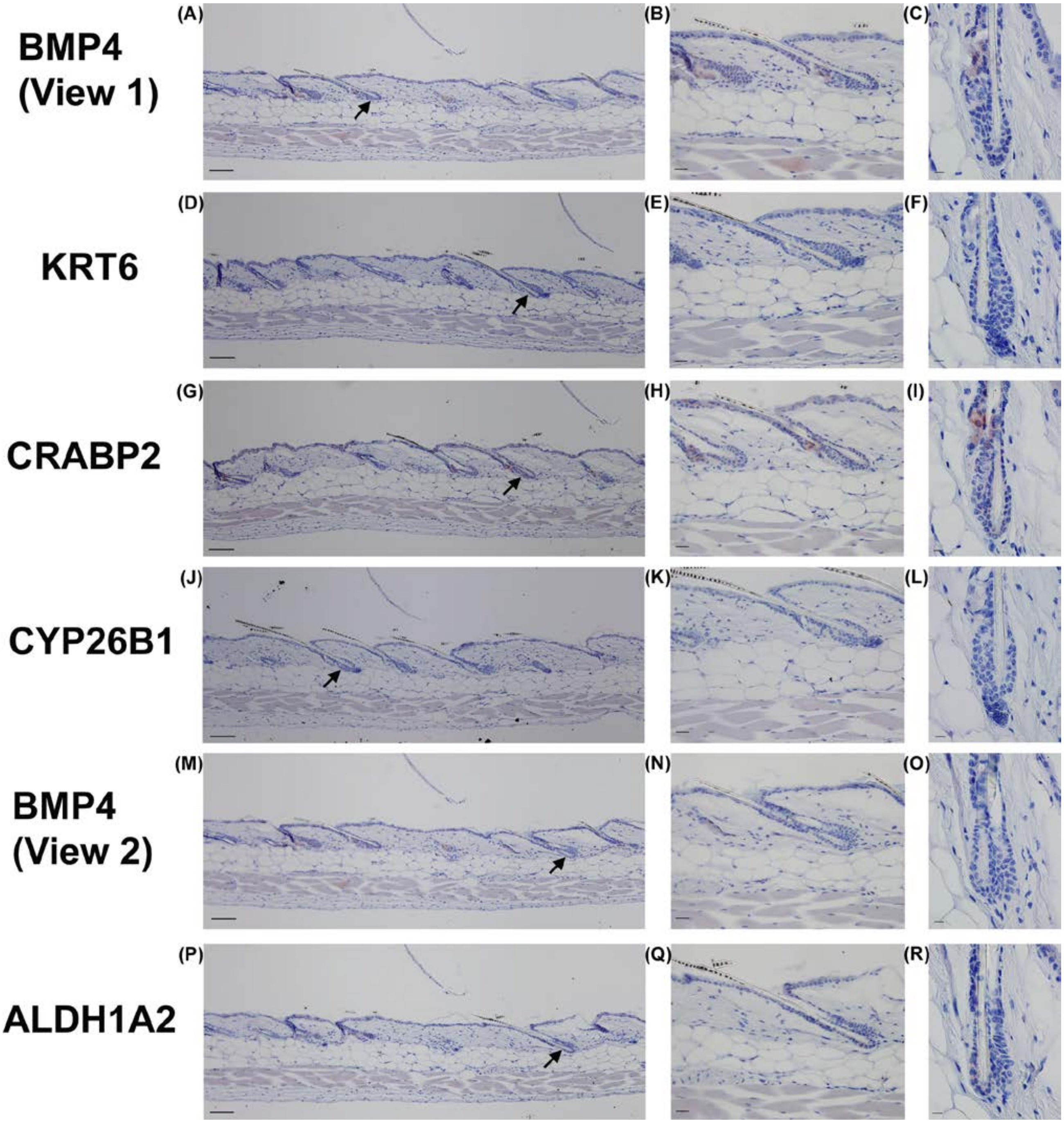

Supplemental Figure 4: Alignment of BMP4 negative hair follicles with vitamin A metabolism proteins. Skin from female C57BL/6J wild type mice was collected at 70-101 days of age (n=1-5

mice/time point). One to three regions of skin were collected per mouse. Immunohistochemistry (IHC) was performed on serial sections with antibodies against BMP4 (A-C, M-O), KRT6 (D-F), CRABP2 (G-I), CYP26B1 (J-L), and ALDH1A2 (P-R) using a red chromagen. An average of 60 hair follicles were examined per region of skin. Bar = 100 um in A, D, G, J, M, and P; Bar = 25.2 um in B, E, H, K, N, and Q, and Bar = 10.1 μm in C, F, I, L, O, and R. Arrow points to the hair follicle used in the higher magnifications. The hair follicles with KRT6, CRABP2, and CYP26B1 match the hair follicle with BMP4 view 1. The hair follicle with ALDH1A2 matches the hair follicle with BMP4 view 2.

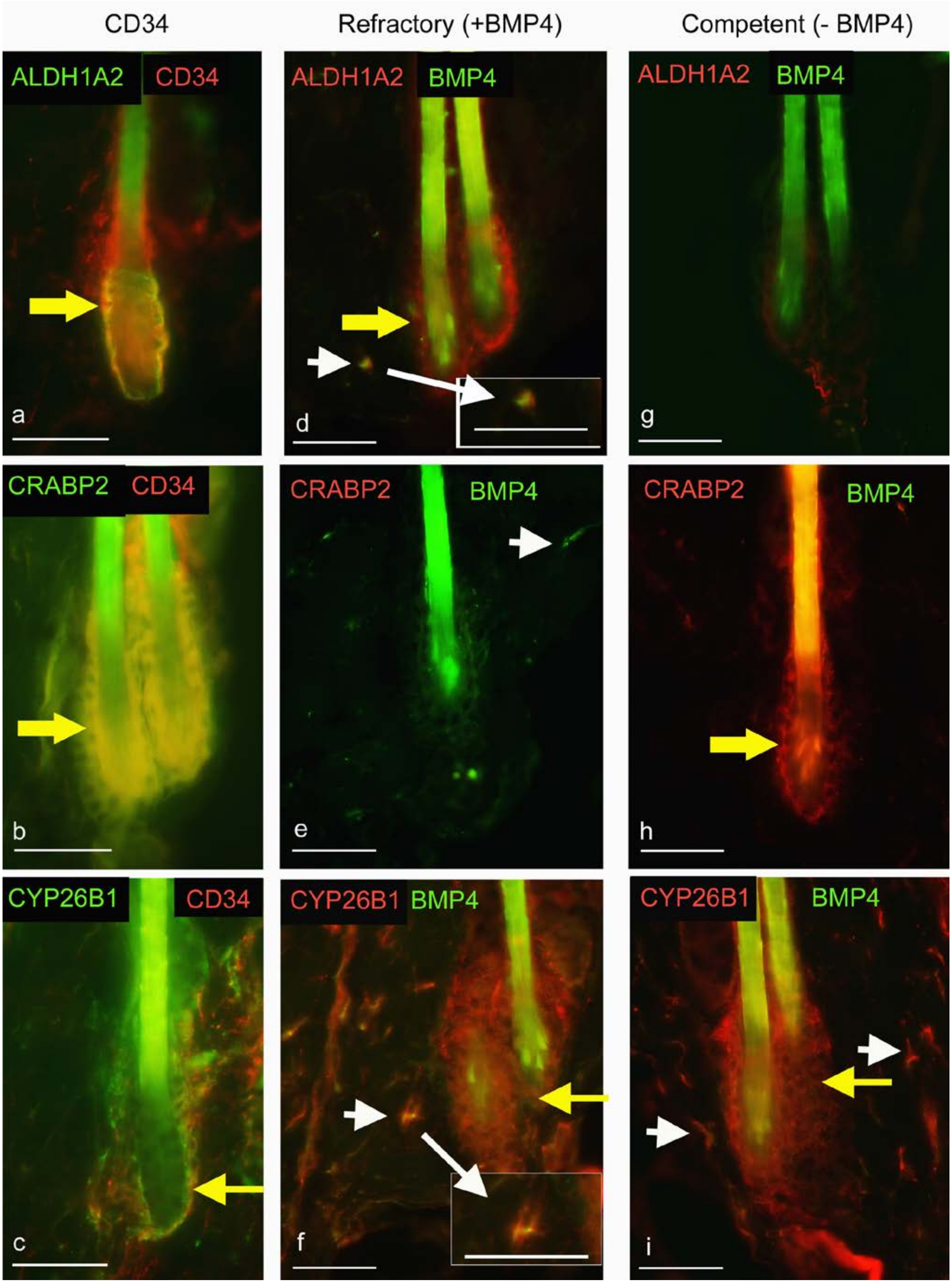
CD34
Refractory (+BMP4)
Competent (- BMP4)
ALDH1A2
CD34
a
ALDH1A2
BMP4
d
ALDH1A2
BMP4
g
CRABP2
CD34
b
CRABP2
BMP4
e
CRABP2
BMP4
h
CYP26B1
CD34
c
CYP26B1
BMP4
f
CYP26B1
BMP4
i

Supplemental Figure 5: ALDH1A2, CRABP2, and CYP26B1 co-localized with hair follicles and was expressed differently in refractory and competent telogen. Frozen skin from six female C57BL/6J mice from the two diet studies were used. Immunofluorescence (IFA) was performed with antibodies against stem cell marker CD34 (red) and ALDH1A2, CRABP2, or CYP26B1 (green) in telogen hair follicle (a-c). To determine the telogen stage of expression, IFA was performed with antibodies against BMP4 (green) and ALDH1A2, CRABP2, or CYP26B1 (red) in telogen hair follicles during refractory (d-f) and competent (g-i) telogen. Bar = 16.6 μM. Yellow arrow = club hair. White arrow = dermal cells.

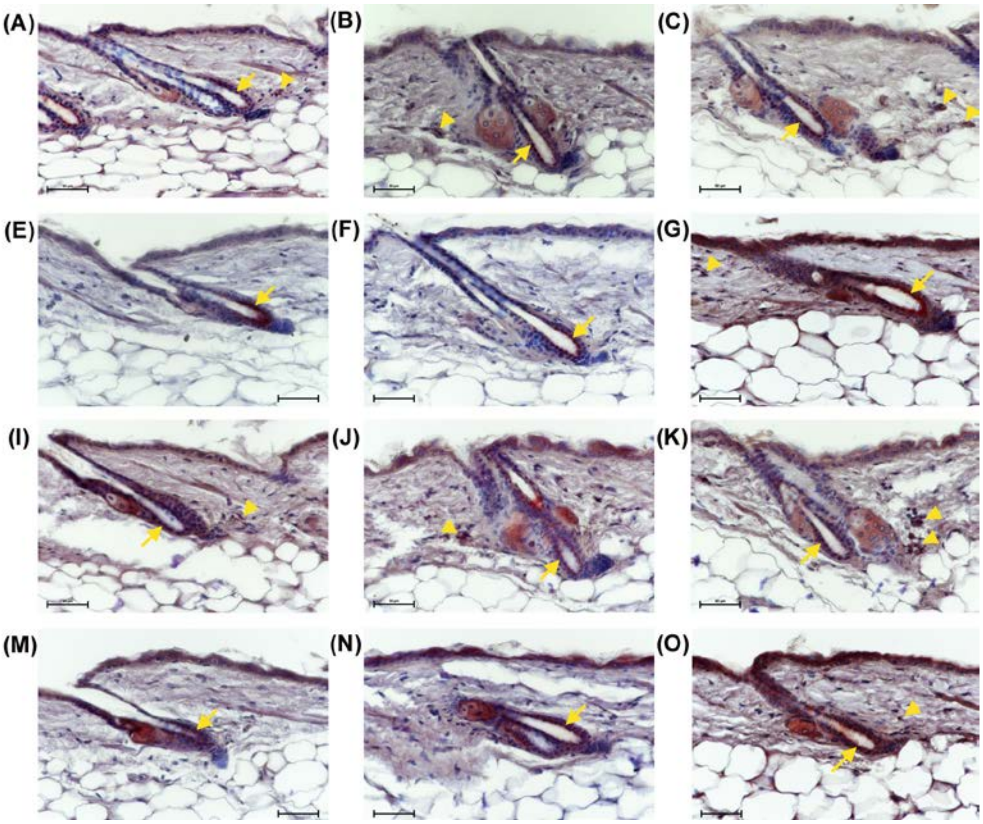

Supplemental Figure 6. Breeding mice on AIN93G diet altered the localization pattern of KRT6 and CRABP2 unaltered images. Study 1 (A-D, I-L, circles) or study 2 (E-H, M-P, squares) mice were switched to the AIN93M diet containing 4 (A, E, I, M n=4-5), 28 (B, F, J, N, n=5), or 56 (C, G, K, O, n=5-7) IU retinyl acetate/g diet. Co-Immunohistochemistry (IHC) was performed with antibodies against KRT6 and CRABP2 (red) with BMP4 (brown, Vector) in skin containing telogen hair follicles. Bar = 50 um. Yellow arrow = inner bulge cells, yellow arrowhead = strong BMP4 positive dermal cells.
